# Human-centred design bolsters vaccine confidence in the Philippines: results of a randomised controlled trial

**DOI:** 10.1136/bmjgh-2023-012613

**Published:** 2023-10-21

**Authors:** Mark Donald C Reñosa, Jonas Wachinger, Jerric Rhazel Guevarra, Jhoys Landicho-Guevarra, Mila F Aligato, Vivienne Endoma, Jeniffer Landicho, Thea Andrea Bravo, Carol Malacad, Maria Paz Demonteverde, Catherine Silvestre, Kate Bärnighausen, Till Bärnighausen, Rachel P Chase, Shannon A McMahon

**Affiliations:** 1 Heidelberg Institute of Global Health, Ruprecht-Karls-Universität Heidelberg, Heidelberg, Germany; 2 Department of Epidemiology and Biostatistics, Research Institute for Tropical Medicine - Department of Health, Muntinlupa City, Philippines; 3 School of Public Health, University of the Witwatersrand, Johannesburg-Braamfontein, South Africa; 4 Africa Health Research Institute, Durban, South Africa; 5 College of Medicine, The Ohio State University, Columbus, Ohio, USA; 6 International Health Department, Johns Hopkins University Bloomberg School of Public Health, Baltimore, Maryland, USA

**Keywords:** vaccines, intervention study, public health, child health

## Abstract

**Background:**

The public’s confidence in vaccinations has eroded, and anti-vaccination movements have gained traction around the world, including in the Philippines. ‘Salubong’, a Filipino term, refers to welcoming someone back into one’s life and elicits ideas about friendship and family relationships. We extended this concept to vaccines in efforts to design an intervention that would re-welcome vaccines into homes.

**Methods:**

Using human-centred design, we developed and refined a story-based intervention that engages Filipino families, community leaders and community health workers. We conducted a randomised controlled trial among 719 caregivers of small children to test the developed intervention against a control video. We assessed the binary improvement (improvement vs no improvement) and the amount of improvement in vaccine attitudes and intentions after intervention exposure.

**Results:**

Although the intervention group began with marginally higher baseline vaccine attitude scores, we found that 62% of the intervention group improved their vaccine attitude scores versus 37% of the control group (Fisher’s exact, p<0.001). Among individuals whose scores improved after watching the assigned video, the intervention group saw higher mean attitude score improvements on the 5-point scale (Cohen’s d=0.32 with 95% CI 0.10 to 0.54, two-sided t-test, p<0.01). We observed similar patterns among participants who stated that they had previously delayed or refused a vaccine for their child: 67% of 74 in the intervention group improved their vaccine attitude scores versus 42% of 54 in the control group (Fisher’s exact, p<0.001). Among the subset of these individuals whose scores improved after watching the assigned video, the intervention group saw higher mean attitude score improvements on the 5-point scale that were marginally significant (Cohen’s d=0.35 with 95% CI −0.01 to 0.70, two-sided t-test, p=0.06).

**Conclusions:**

Our results provide solid evidence for the potential of co-designed vaccine confidence campaigns and regulations.

**WHAT IS ALREADY KNOWN IN THIS TOPIC**

- Vaccine hesitancy can have serious public health consequences, as it can lead to outbreaks of vaccine-preventable diseases, particularly in countries with low vaccination rates.
- Concerns about the safety and effectiveness of vaccinations, including misinformation, mistrust of healthcare workers or pharmaceutical companies, religious or philosophical convictions and fear of side effects are all potential causes of vaccine hesitancy.
- While human-centred design (HCD) has proven beneficial in several health campaigns, evidence regarding whether, how or to what effect HCD can be used to bolster vaccination confidence in low and middle-income countries is lacking.

**WHAT THIS STUDY ADDS**

- We designed and refined a story-based intervention that involves Filipino families (especially those who are vaccine-hesitant), community leaders and community health workers using HCD.
- Our findings highlighted the potential of real-life narratives in developing and honing an intervention rooted in the local context.
- Our HCD-driven intervention boosts vaccine confidence and increases positive feelings about vaccines. We thereby reinforce the importance of HCD as a method of meaning-making that affects attitudes and behavioural intent in relation to vaccinations.

**HOW THIS STUDY MIGHT AFFECT RESEARCH, PRACTICE OR POLICY**

- Time, vulnerability and volatility (ie, roller-coaster emotions) elements of vaccine hesitancy emphasise the necessity of integrating context and ongoing public sentiments into interventions targeted at promoting vaccine confidence.
- Additional and larger-scale research is warranted, particularly concerning vaccine messaging revitalisation in a time of pervasive disinformation and with vaccine uptake outcomes in addition to intentions.

## Introduction

The need for interventions and products that are personalised to human experiences and their cultural and environmental contexts is increasingly recognised. Human-centred design (HCD) has gained popularity in the field of global health as a means to co-create and rapidly assess products and services.^1 2^ HCD engages intended end-users in the design process and encourages implementers to be guided by empathy when developing applied solutions.^1^ In prioritising end-user insights and engaging with end-users throughout design ideation and iterations, HCD cultivates a sense of equity.^3^

Several studies have demonstrated the value of HCD in fostering cultural sensitivity and local adaptability.^4–8^ HCD-driven breastfeeding interventions in South Africa, for example, emphasised the relevance of stories and personal experiences, as well as leveraging local talents and expertise (ie, elements of local voices and music) to resonate across ethnic and socioeconomic groups.^5 8^ The development of a maternal nutrition video in Burkina Faso involved modifying images related to climate, language, food, household structures and socioeconomic position of end-users and healthcare professionals as a means to enhance relatability.^6 7^ In India, HCD was used to generate digital health solutions (eg, mobile messaging service) for long-term health system integration to address maternal and child health in the country.^9^ Additionally, several HCD-driven interventions have focused on the use of straightforward storylines and journey maps when designing products.^4 10^

As vaccine hesitancy constitutes a significant threat to global health,^11^ HCD has proven to be beneficial in developing vaccine promotion material.^12–16^ Examples include development of user-centred mobile applications to educate parents on child vaccination and vaccine safety communication strategies in Germany,^13 16^ paediatric vaccination modules for patients in Argentina^14^ and clinical guidelines to improve healthcare providers’ recommendations in the USA.^15^ Some less-resource settings have also started to incorporate HCD principles into their work, such as the development of educational materials and interactive elements to engage parents in South Africa and Burkina Faso,^4–8^ hospital-based vaccine documentation strategies in Kenya^10^ and digital interventions in India.^9 12^ Existing vaccine confidence interventions employing HCD rely heavily on health-system approaches and advocate for improved healthcare environments.^12–16^

While HCD-driven interventions have yielded promising results in terms of increasing vaccine confidence and uptake, their scope did not always acknowledge vaccine-hesitant families’ own lived experiences and narratives. Several authors have argued about the importance of tailoring health interventions based on vaccination concerns and experiences, and aligning interventions to fit cultural and environmental contexts.^17 18^ More recently, authors have also highlighted a need to address hesitancies rooted in alternative health beliefs, political polarisation or belief-based extremism reinforced by digital media platforms.^18^ Additionally, despite increasing in recent years, scholarship from low and middle-income countries on employing HCD for the development of vaccine confidence interventions remains limited.

To fill gaps in the literature and lay the groundwork for a meaningful campaign that restores trust in vaccines, we drew on local narratives to design, refine and ultimately test a story-based intervention that connects vaccine-hesitant caregivers (eg, parents, other family members, legal guardians), policymakers, healthcare workers (HCWs) and other community actors. We developed and tested our HCD-driven intervention in a country that has experienced an unprecedented erosion of vaccine confidence in childhood vaccinations: The Philippines. Dramatic declines in vaccine confidence and uptake in the Philippines are linked to a dengue vaccination controversy in 2017, which sparked widespread distrust in childhood vaccinations and led to large-scale measles outbreaks and the loss of a 20-year polio-free status in 2019.^19 20^ Against this contextual backdrop, we developed an animated video intervention called ‘Salubong’, a Filipino term that refers to welcoming someone back into one’s life and which elicits ideas about friendship and family relationships. We extended this concept to vaccines to design an intervention that would encourage re-welcoming vaccines into homes.^21^

In this article, we present the randomised controlled trial (RCT) results of testing the final story-based vaccine confidence intervention. Our work provides evidence that can inform upcoming campaigns and regulations targeted at restoring public confidence in vaccines.

## Methods

### Study design and setting

We undertook an RCT targeting parents or caregivers of under-five children in urban and rural communities of Calabarzon region, the Philippines. Calabarzon region (population ~16 million) is the most populous region in the Luzon group of Islands, where measles cases rose 300% in 2019.^22–24^ We purposively selected Dasmariñas City (urban arm) and Silang, Cavite (rural arm) to reflect both rural and urbanised conditions, and to capture different and varied sociodemographic factors and health facility-related experiences on child health and vaccinations. A published protocol and methodological articles provide a detailed overview of the study design and data collection techniques.^21 25 26^

Table 1 shows the summary of the study phases, specific objectives and corresponding outputs, which track the four phases of HCD. Within any given phase, we allowed for iterations and repetitions as necessary. We had to forgo in-person data collection due to the COVID-19 pandemic^25^ and shifted all data collection activities to a remote RCT in line with the procedures outlined in the published protocol^21^ and the recommendations of the European Medicines Agency and the Philippines Inter-Agency Task Force amid COVID-19 pandemic to ensure study participants’ and researchers’ protection. Data collector trainings included modules on computers, apps, video conferencing platforms and online voice recorders, as well as data backup and security protocols.^25^ We used Zoom breakout rooms to train data collectors, which allowed them to practice survey approaches in different groups, with and without trainer supervision. For consenting, in lieu of meeting participants in-person and establishing informed consent by signature or fingerprint, participants signed consent forms remotely during a recorded video call and shared a ‘selfie’ with the signed form.^25^

**Table 1:** Details of the development of ‘SALUBONG’ intervention video

Date	Human-centred design component	Aims and target groups	Outputs
August–September 2020	Preparatory phase	Understanding the challenges on childhood vaccinations, perceptions of vaccines and health system In-depth interviews (IDIs) with policymakers (n=19)	Narratives and descriptions
October–December 2020	Phase 1: Shared Appraising: ‘Empathise’	Gathering information about how participants frame vaccine hesitancy as a problem, how they situate themselves (particularly considering their sociocultural context) and learning which factors would motivate them to address the problem IDIs with vaccine-hesitant caregivers (n=44), vaccine-accepting caregivers (n=11), healthcare workers (n=7) and community leaders (n=3)	Storyboard development
January–March 2021	Phase 2: Life stories and Uncovering: ‘Define and Ideate’	End-users suggest ideas to address the problem in collaboration with the research team Focus group discussions (FGDs) (n=5) and IDIs (n=6) with caregivers and FGDs with community health workers (n=4)	Refined storyboards
April–July 2021	Phase 3: Bridging and Optimising: ‘Prototype’	Prototypes and products are then developed and tested in real-world settings with actual users via actual delivery systems IDIs with healthcare workers (n=14)	Co-produced prototype (5 min animated cartoon video+online delivery approach)
August 2021–August 2022	Phase 4: Navigating and Gaining: ‘Test’	A particularly promising product is introduced more broadly Randomised controlled trial with caregivers (n=719)	Tested intervention

**Figure 1:**
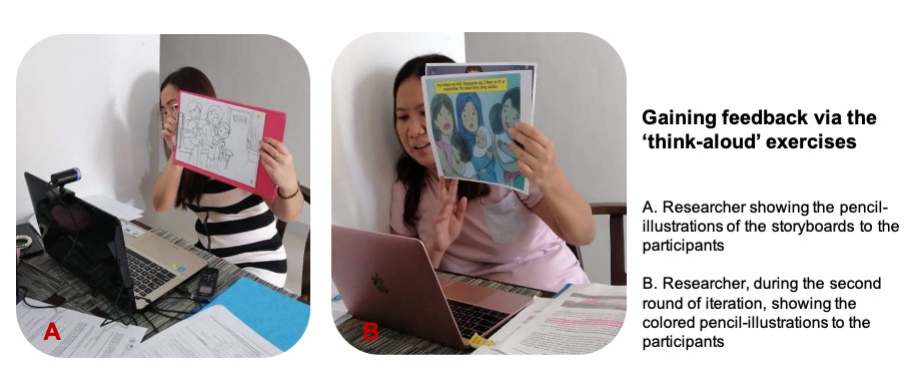
Think-aloud exercises performed during the iteration and prototyping phase.

The stories and experiences of participating vaccine-hesitant caregivers served as the impetus for the Salubong video.^27 28^ Qualitative data collected amid HCD phases outlined the role of sociocultural context in shaping vaccine hesitancy in the Philippines and the widespread consequences of the dengue vaccine scare across various population strata. These findings highlighted the potential of real-life narratives in developing and honing an intervention rooted in the local context.^27–29^ Preliminary cartoon sketches and characters for the storyboards were presented and iterated along the way. We performed think-aloud exercises with caregivers, HCWs and community leaders using analogue flipboards and/or screen share digital photos of the paper-based storyboards to critique and refine the storyboards (see figure 1). Local Filipino cartoonists were involved throughout the design process, including via: (a) sharing of video snippets of qualitative interviews including design feedback; (b) collaboration in debriefings and/or provision of the debriefing notes; (c) consultative Zoom meetings on the development and refinement of the video storyboards and (d) continuous refinement of the sketches based on emerging design insights. We had originally planned to have local cartoonists join in on the online interviews as observers, but we ultimately decided against it because of the sensitivity of the vaccination topic in the local context and ethical considerations.

**Figure 2:**
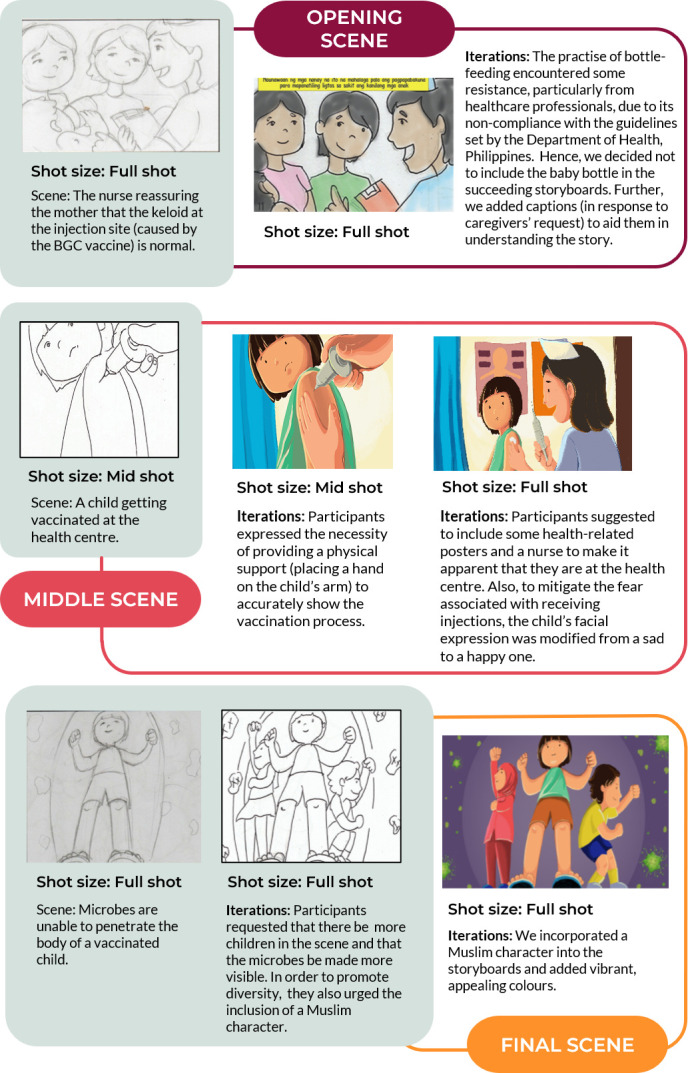
Iterations—before and after adaptation—of the SALUBONG intervention.

The HCD-driven intervention was iteratively improved based on participants’ feedback and insights from policymakers and various actors working directly with the public health system to counteract falling immunisation rates.^29^ Figure 2 shows the preliminary results of these iterative and prototyping processes. The full complexities of the design process, which lasted 12–15 months, entailed extensive discussions among various actors (scientists, policymakers, healthcare providers, community and local stakeholders, animators and cartoonists, health promotion experts, communication and social media officers, etc) and further details of the processes in terms of how we chose the medium and the message will be presented elsewhere.

**Figure 3:**
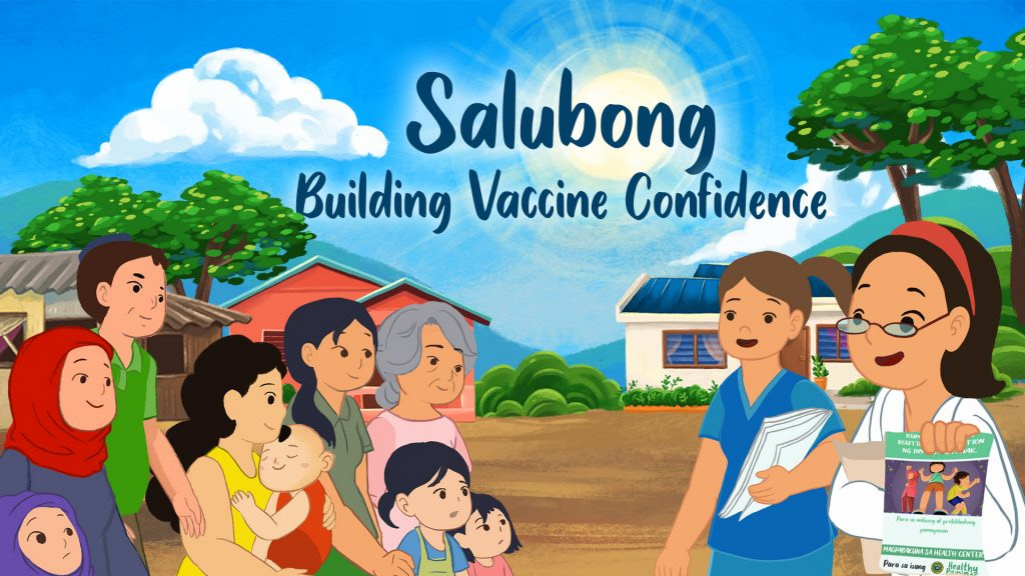
Cover of the cartoon ‘SALUBONG: Building vaccine confidence’.

The intervention video included a 5-minute animated cartoon, entitled: ‘Salubong: Building Vaccine Confidence’, that narratively featured stories of Filipino families about vaccines (the front cover of the video is shown in figure 3). The cartoon used a narrative and empathic format tailored to the Filipino cultural setting, featuring diverse characters of different ages, household compositions and income, and ethnic backgrounds, as well as appealing colours for optimal contrast. The Salubong video was co-created in collaboration with study participants, and Filipino local cartoonists and dubbed by voice actors from The Coffee Creatives, an animator’s studio in the Philippines. The original video is in Filipino, and there are two other versions with Filipino and English subtitles. The Salubong intervention video is available for viewing (https://www.youtube.com/watch?v=M8nEj5G9Iuc).

### Study oversight

Prior to commencement, permission to undertake this study was obtained from Department of Health officials (national, regional and provincial offices). Further, through official letters and Zoom courtesy calls, we also acquired permission from local authorities and leaders in the Calabarzon region to carry out the study in their respective communities. Informed consent was obtained from all participants prior to their enrolment.^25^

### Experimental design and set-up

We randomly selected barangays (‘small communities’) from Dasmariñas City (urban arm) and Silang, Cavite (rural arm) that had not previously participated in a qualitative component of the study (results of which are described elsewhere^27 28^). We estimated sample sizes of 200 participants per group (intervention and control arms in rural and urban areas), yielding a total of 800 individuals. With an 85% response rate, a 5% type I error rate, and a 20% type II error rate, the calculated sample size allowed us to detect a difference of 15% in the binary outcome between the intervention and control groups in each area.

We performed a multistage stratified sampling frame to select barangays. From each of the two study sites, two barangays with the highest population, based on the most recent population report available, were selected to ensure enough potential participants. The two selected barangays (per urban and rural arms) were randomly allocated as an intervention and as a control site. A listing of households with under-five children was obtained from the local health officials. The household listing served as a sampling frame from which 200 households were randomly selected and invited to participate in the study. In four originally sampled barangays (two each for control and intervention groups), the number of interviewed caregivers was less than 200. In line with the processes defined in the research protocol, we therefore sampled additional barangays as per the criteria above.

We collaborated with community health workers who conducted house visits, obtained caregivers’ mobile phone numbers and obtained consent to be contacted by the research team, following the selection and allocation of the potential participants to intervention or control groups. Community health workers also distributed consent forms and informed potential participants that a member of the research team would contact them. Afterwards, the potential participants were invited to participate during a phone call where the study aims were briefly introduced. If individuals expressed interest in the initial phone contact, we either directly continued with a detailed study discussion and obtained online informed consent (via Facebook Messenger video call) or scheduled a separate appointment. To ensure participants’ internet connectivity throughout the consent process and trial procedures, we purchased and transmitted mobile data packages to participants.

Once consent was obtained, the videos (intervention and control) and surveys (pre and post) were delivered online. We developed and used an online version of the survey forms, which we pilot-tested among 30 caregivers to ensure feasibility (either self-administered or data collector-assisted) and alleviate operational challenges. Following the pilot testing of the survey forms, we therefore decided to conduct data collector-assisted surveys (ie, data collectors read the questions to the participants and are responsible for encoding the answers in the online form). After completion of the baseline survey, the Salubong video was then screened for members belonging to the intervention group, while the ‘*Paano labanan ang COVID-19* (How to fight COVID-19)’ animated video, which was created by the Philippines Department of Health’s Health Promotion Bureau (ie, focused on the value of staying at home, transmission, and signs and symptoms), was screened for control group participants. We used ‘online screen-sharing’ functions to screen videos for participants. To avoid biases or contamination of the data, no interactions were attempted during the presentation of the videos. Following the video, we conducted a follow-up using the same survey as the baseline assessment (without re-capturing the sociodemographic information gathered in the baseline survey).

Before and after watching the intervention or control video, participants in both groups were asked to provide information about their attitudes toward vaccinations, with statements such as, ‘Children get more shots than are good for them’, ‘I believe that many of the illnesses that vaccinations prevent are severe’ and ‘It is better for my child to develop immunity by getting sick than to get a shot’, among others. We used the parents’ attitudes about childhood vaccination (PACV-15) adapted from Opel and colleagues.^30–34^ The PACV-15 exhibits robust psychometric properties^30–34^ and has proven to be a valuable instrument extensively employed in prior research assessing parental vaccine attitudes.^35 36^ While we left the fundamental structure of the tool unchanged, we made modifications to the questionnaire wording to align with the study context and retained more of the granularity of 5-point and 10-point Likert items when scoring compared with the collapsed category scoring of the original PACV-15 calculation method. This allowed us to focus primarily on how the intervention affected response patterns and to assess whether the intervention caused, for example, polarisation of attitudes while enabling us to make the cultural comparisons and gain the insights into parental attitudes towards vaccines that the PACV-15 allows. The full questionnaire is included as online supplemental file 1. Responses ranged from ‘strongly disagree’ to ‘strongly agree’ on a 5-point scale. To lessen the subjective uncertainty associated with Likert Scales, we presented the responses as visual analogues (ie, a combination of facial emojis, colour gradations, numbers and integration of Filipino words).

10.1136/bmjgh-2023-012613.supp1 Supplementary data

### Data treatment and analysis

Achieving the originally envisioned sample size proved challenging. As outlined above, we sampled participants from more barangays than initially envisioned, (a total of 8 in control and 4 intervention barangays across urban and rural study sites), following the pre-defined procedures, due to difficulties reaching participants fulfilling the inclusion criteria under pandemic conditions, and as many potential participants refused to participate online. We considered shifting to in-person data collection but ultimately decided against it due to the COVID-19 concerns raised by the selected barangays and budgetary constraints. Instead, we analysed the data sets available after more than 1 year of data collection (n=719) to see if adding more information could impact the primary outcomes. We calculated whether knowledge had increased in total (ie, the total points in the suggested approach are higher in pre-intervention and post-intervention) and in which particular domain knowledge had increased. Furthermore, we did linear regression analysis with ‘change in scores’ as the result, ‘intervention’ as the major factor and ‘demographics’ as the other components. As there was little room for improvement via the intervention because of the high percentage of participants in the intervention and control groups expressing the desired response to D1 (‘delays in taking vaccines’), we concluded that additional sampling to include the originally envisioned n=800 participants could not change the study’s conclusions with regard to this outcome. Secondary outcomes would similarly be unaffected by additional data collection. We therefore stopped collecting data and analysed the available data sets.

Overall, we assessed the binary improvement (improvement vs no improvement) and the amount of improvement in vaccine attitudes and intentions after intervention exposure. The PACV-15 Likert Scales were labelled D4–D13 (10 items), and the responses were transformed from a 1 to 5 scale to a −2 to 2 scale, with −2 representing the least desired option (regardless of whether that was 1 or 5 in the original scale). The more items we included, the more granular the differences we could discern both person-to-person and within a person over time. Additionally, the Likert scale responses were analysed inferentially by coding answers from 1 (strongly disagree) to 5 (strongly agree) and drawing on parametrical (paired t-test) or non-parametrical (Wilcoxon signed rank test) approaches, depending on sample characteristics. Electronic copies of all data were saved offline and external hard drives were stored in a locked cabinet at the Research Institute for Tropical Medicine in the Philippines. All data management and analyses were performed using STATA (Stata Corp, College Station, Texas, USA) and R (R Development Core Team, Vienna, Austria) statistical software. An author reflexivity statement on our partnership is included as online supplemental file 2.

10.1136/bmjgh-2023-012613.supp2 Supplementary data

### Patient and public involvement

Patients and the public were not directly involved in the design, conduct, reporting, or dissemination plans for this study. However, the research team consistently gathered participant narratives and feedbacks in accordance with the principles of HCD (for the overall study) and qualitative research, and the findings provided here give voice to these participant experiences.

## Results

### Demographic characteristics and participant flow

**Figure 4:**
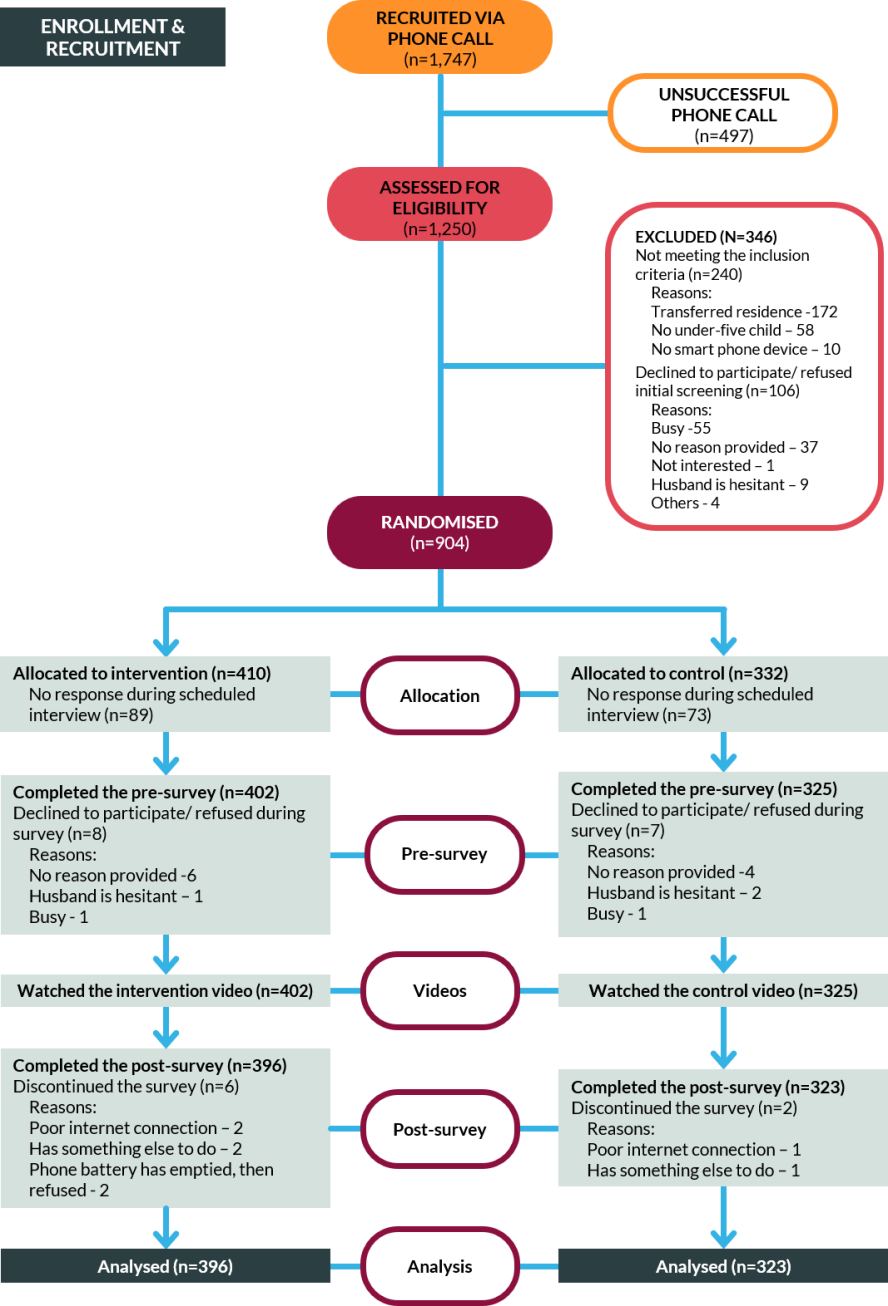
Trial recruitment and retention of participants.

Between 11 August 2021 and 15 August 2022, 719 participants were surveyed; 396 participants were from urban areas, while 323 were from rural areas (table 2). A majority of participants were women (96%), were most frequently housewives (51%) and had completed at least their high school education (79%). Among the sociodemographic characteristics, only the place of residence differed significantly between intervention and control groups (Pearson χ=11.10, p=0.001). After receiving a randomly assigned treatment, 396 participants watched the Salubong video while 323 participants saw a standard COVID-19 health education video. Figure 4 shows a diagram of the participant flow.

**Table 2:** Sociodemographic characteristics

Characteristics	Total (n=719)	Intervention (n=396)	Control (n=323)
Sex, n (%)			
Female	693 (96.4)	379 (95.7)	314 (97.2)
Male	26 (3.6)	17 (4.3)	9 (2.8)
Age, mean (SD)	32.7 (8.7)	33.0 (8.9)	32.2 (8.5)
Place of residence, n (%)			
Rural	396 (55.1)	196 (49.5)	200 (61.9)
Urban	323 (44.9)	200 (50.5)	123 (38.1)
Participant’s occupation, n (%)			
Housewife	364 (50.6)	207 (52.3)	157 (48.6)
None	113 (15.7)	59 (14.9)	54 (16.7)
Business	82 (11.4)	46 (11.6)	36 (11.1)
Manual labourer	50 (7.0)	22 (5.6)	28 (8.7)
Self-employed	49 (6.8)	30 (7.6)	19 (5.9)
Professional	46 (6.4)	26 (6.6)	20 (6.2)
Community health worker	8 (1.1)	3 (0.8)	5 (1.5)
Student	4 (0.6)	2 (0.5)	2 (0.6)
Clerical support	1 (0.1)	0 (0.0)	1 (0.3)
Retired	2 (0.2)	1 (0.3)	1 (0.3)
Educational attainment, n (%)			
None	2 (0.3)	2 (0.5)	0 (0.0)
Elementary	51 (7.1)	33 (8.3)	18 (5.6)
High school undergraduate	97 (13.5)	47 (11.9)	50 (15.5)
High school graduate	304 (42.3)	176 (44.4)	128 (39.6)
Vocational education	51 (7.1)	27 (6.8)	24 (7.4)
College undergraduate	120 (16.7)	63 (15.9)	57 (17.7)
College graduate	90 (12.5)	47 (11.9)	43 (13.3)
Graduate studies	4 (0.6)	1 (0.3)	3 (0.9)
Primary healthcare decision-maker in the family, n (%)			
Mother	540 (75.1)	296 (74.7)	244 (75.5)
Father	111 (15.4)	61 (15.4)	50 (15.5)
Grandmother	55 (7.6)	33 (8.3)	22 (6.8)
Grandfather	5 (0.7)	2 (0.5)	3 (0.9)
Other sibling	3 (0.4)	0 (0.0)	3 (0.9)
Both parents	2 (0.3)	1 (0.3)	1 (0.3)
Both grandparents	2 (0.3)	2 (0.5)	0 (0.0)
Live-in partner	1 (0.1)	1 (0.3)	0 (0.0)
Number of under-5 years old children per household, mean (SD)	1.3 (0.5)	1.3 (0.5)	1.3 (0.5)
Number of all children per household, mean (SD)	2.4 (1.3)	2.4 (1.3)	2.3 (1.3)

### HCD video increases vaccine confidence

**Figure 5:**
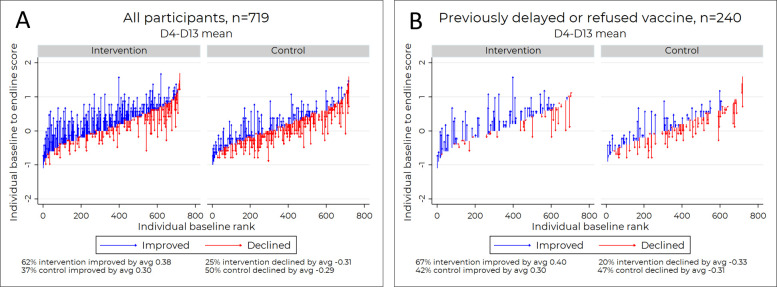
Caregiver’s attitudes toward childhood vaccines pre-intervention and post-intervention.

Figure 5A depicts a positive upward trend in the mean improvement on D4–D13 after exposure to the intervention video versus the control video. Although the intervention group began with marginally higher baseline vaccine attitude scores, our findings showed that 62% of the intervention group improved their vaccine attitude scores versus 37% of the control group (Fisher’s exact, p<0.001), and the mean attitude score improvements on the 5-point scale were higher when limiting assessment to those whose scores improved after watching the assigned video (Cohen’s d=0.32 with 95% CI 0.10 to 0.54, two-sided t-test, p<0.01). Comparing the intervention group to the control group, participants in the intervention group were substantially less likely to experience post-test mean score reductions, but among those who did, there was no statistically significant difference in score change (−0.31 vs −0.29, two-sided t-test, p=0.34).

When limiting the analysis to participants who had previously delayed or refused vaccination in the past, we were left with 240 participants, some in intervention and some in the control groups. Participants who stated they had previously delayed or rejected vaccinating their children exhibit similar patterns: 67% of 110 in the intervention group improved their vaccine attitude scores compared with 42% of 130 in the control group (Fisher’s exact, p<0.001) (figure 5B). Among individuals whose scores improved after watching the assigned video, the intervention group saw higher mean attitude score improvements on the 5-point scale that were marginally significant (Cohen’s d=0.35 with 95% CI −0.01 to 0.70, two-sided t-test, p=0.06). Intervention participants (who had previously delayed or refused vaccination) were much less likely to see declines in their mean scores post-test compared with control, but among those who did decline, there was no statistically significant score change when comparing intervention versus control (−0.33 vs −0.31, two-sided t-test, p=0.73). Further, the participants (who had previously delayed or refused vaccination) were substantially more likely to increase their score at all, and those in the intervention group who did so marginally more than those in the control group.

### Intervention showed vaccine confidence score improvements among those who did not trust HCWs

**Figure 6:**
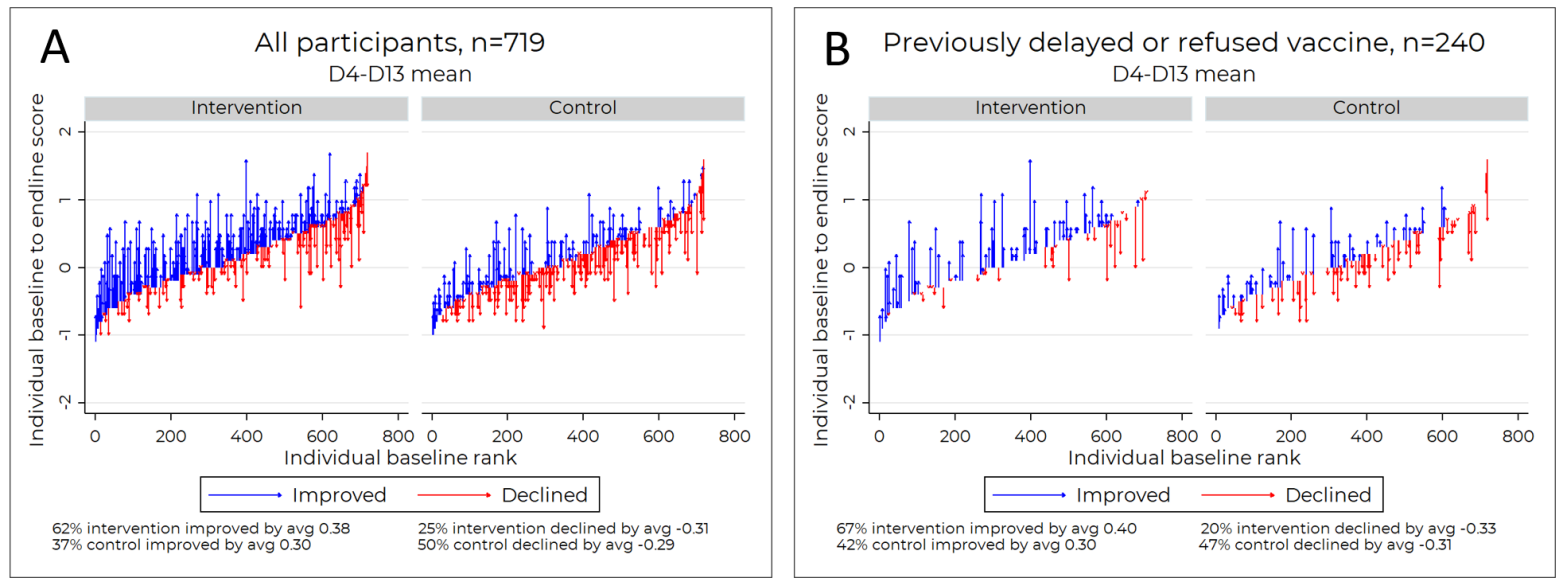
Caregiver’s attitudes regarding HCWs pre-intervention and post-intervention. HCWs, healthcare workers.

Participants who listed HCWs as among their most trusted sources of vaccine information had baseline average scores that were higher than those who did not (0.15 vs 0.03; t-test, p=0.02) (figure 6). When analysing intervention versus control score changes from pre-test to post-test in each group determined by HCW trust status, intervention had greater score improvement than control insofar as more intervention group participants saw improvements in their post-test versus pre-test scores (66% vs 28% among those who did not trust HCWs; 61% vs 39% among those who did trust HCWs), but among those whose scores improved, the amount of improvement was similar comparing intervention to control (0.35 vs 0.29 among those who did not trust HCWs; 0.38 vs 0.30 among those who did trust HCWs).

**Figure 7:**
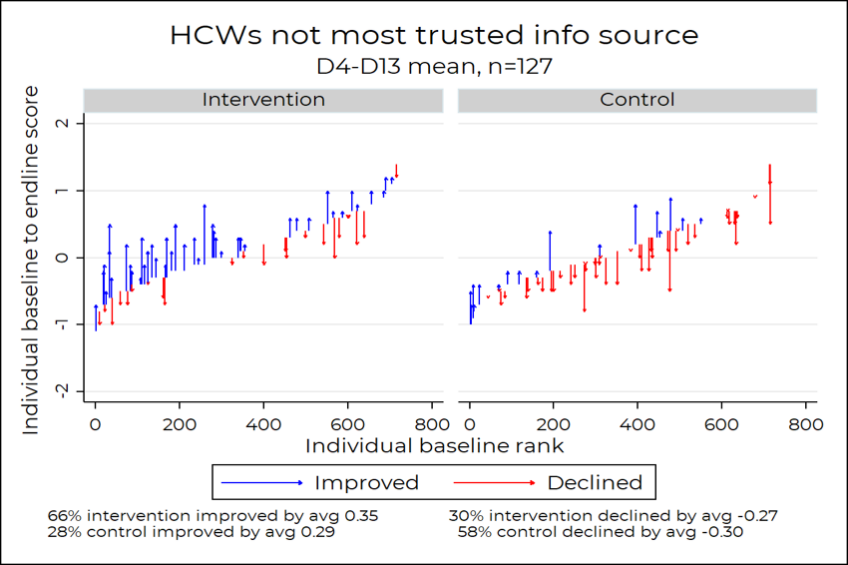
Caregiver’s who do not list HCWs as their most trusted source of information. HCWs, healthcare workers.

We narrowed our analysis to people whose most trusted source of information was someone other than HCWs, as they are an important group to reach with vaccine confidence messaging and they had a significantly different baseline score than others (figure 7). Our results showed that the intervention group still reflected statistically significant improvements in post-test scores compared with the control group in terms of more intervention group participants’ scores improving (66% vs 28%, Fisher’s exact p<0.001), with the improvers among the two groups improving a comparable amount (0.35 intervention vs 0.29 among controls, two-sided t-test, p=0.35).

### Human-centred video boosts positive feelings about vaccines

**Figure 8:**
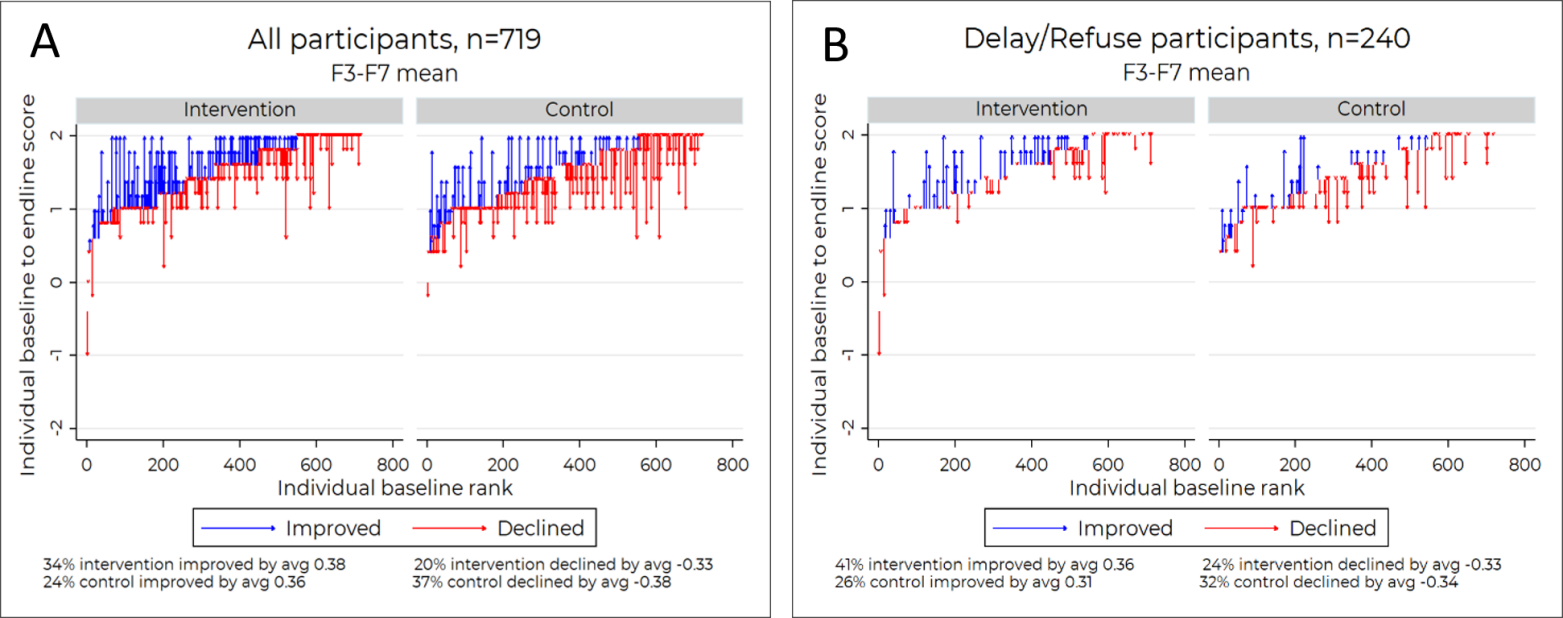
Perception to the intervention and control videos.

We assessed participants’ affective responses to the videos both before and after they watched the intervention or control videos (figure 8). We included declarations like ‘I feel that the people in the healthcare system respect my situation’, ‘I feel that I am warmly welcomed by healthcare workers in the health facilities’, and ‘I feel that agreeing to vaccines is a way to show my love for my children’. The baseline values were extremely high, with an average of 1.47 (with a possible range of −2 to 2); these items therefore had very little space for improvement in a pre-test to post-test comparison. The intervention group, however, still included more people who improved their scores (34% vs 24%) and fewer individuals whose scores declined at post-test (20% vs 37%) than the control group.

## Discussion

To the best of our knowledge, this is the first RCT that examined the efficacy of a video-based, HCD-driven intervention improving parental confidence in childhood vaccinations in the Philippines. We found that our HCD-driven intervention, the Salubong animated video, positively impacted caregiver attitudes and confidence toward childhood vaccinations. Our intervention also improved vaccine confidence among participants who previously delayed or refused a vaccine for their children. Our findings reaffirm the value of HCD as a meaning-making approach that influences attitudes and behavioural intent in general^1 2 37 38^ including in relation to vaccination.^39^

Our results support the use of HCD to boost vaccine confidence along the vaccine hesitancy continuum. A number of research studies have used HCD when developing vaccine confidence interventions with promising results, for example, to design mobile Apps to inform parents about the vaccination status of their children in Germany^13^ and to increase HPV vaccination uptake among adolescent girls in the USA.^40^ Also, UNICEF’s Human Centred Design 4 Health initiative includes several case studies of countries that have implemented HCD-driven interventions including the ‘rock in a jar’ in Mali (which involves giving grandmothers a jar full of stones to ease their burden of remembering vaccination schedules) and a ‘playmat board game’ in Nigeria (which portrays a journey map of critical milestones and health-seeking behaviours to bring health education closer to households).^41^ Our findings add to the growing collection of HCD-driven initiatives for public health promotion to bolster vaccine confidence.

Some studies also provide evidence of the effectiveness of different types of vaccine confidence interventions and highlight the importance of addressing vaccine confidence as a barrier to vaccine uptake. Prior successful interventions include text message reminders for influenza vaccine uptake in Australia,^42^ an internet-based social media intervention to address parents’ vaccine concerns in the USA^43^ and an individually tailored educational application for pregnant women and mothers in the USA.^44^ Our findings contribute to the literature by showing that vaccine-hesitant individuals can improve their vaccine intentions and the potential of HCD-driven interventions in promoting vaccine confidence.

Our findings also provide concrete evidence of the opportunities of empathic-driven interventions, particularly for low-resource settings combating vaccine losses brought on by controversies.^41^ The notion that vaccine hesitancy has time, vulnerability and volatility (ie, roller coaster sentiments) dimensions underscores the importance of incorporating context and ongoing public sentiments in any interventions aimed at boosting vaccine confidence.^18^ Ofri described this phenomenon as ‘emotional epidemiology’, arguing for it to be as critical as clinical epidemiology and calling for addressing the existential concerns of the public over vaccines.^45^ Similarly, in light of the volatility of vaccine hesitancy, Larson urges researchers to reconfigure the definition and measurements as ‘vaccine hesitancy is not a behaviour’, but rather ‘a psychological state of indecisiveness’.^46^ Contextual awareness then is essential in evaluating how much traction a particular vaccination effort can receive and how substantial a reaction it can generate.^18 46^ Therefore, HCD offers a promising mechanism to contend with the volatility inherent to vaccine hesitancy by putting the needs, wants and experiences of people at the centre of the design process. In terms of measuring vaccine hesitancy in our study, we acknowledge PACV-15 as a valuable tool for measuring changes in vaccine confidence.^30 31^ Additionally, we drew on affective questions to emphasise vaccine-related emotional responses to account for vaccine hesitancy’s volatility, which allowed us to better understand the affective responses our participants expressed towards vaccines adding to the ongoing discussion about suitable metrics and additional indicators for capturing the complexities of vaccine hesitancy.

While our findings are generally encouraging, we note limitations. First, confidence and intent to vaccinate do not always translate into actual vaccination uptake. We invite future researchers to implement similar interventions that include actual vaccine uptake as an outcome measure. Second, certain concerns might be associated with this study being conducted online, especially regarding internet connectivity concerns and informed consent processes. However, we developed online data collection and standard operating procedures to allay these operational worries.^25^ Third, we also recognise that survey questions might lead to response biases, especially when they are delivered online; to keep participants focused, we avoided using one single question design throughout the survey. Instead, we used a combination of binary questions, Likert Scales, and response continuums to provide a range of response options and maintain engagement. Additionally, we translated the options into the local language and modified our Likert Scales to incorporate facial emojis, colour continuums, and number continuums. Therefore, by employing these strategies, which urge participants to think about their responses, we hope to have alleviated response biases. By delivering the survey online, we might expect to see less social desirability bias and greater item non-response; while we could not assess for social desirability bias using the data we collected, item non-response rates were zero on both intervention and control. Finally, we draw attention to the fact that a majority of our participants are women. While women are regarded as key caregivers in Filipino households, the lack of other perspectives might have led us to overlook nuances in vaccine decision-making. Therefore, we encourage future research to specifically target diverse household members.

## Conclusion

Our study confirms that HCD is a promising approach to improving vaccine attitudes and intentions. Our findings may help shape future initiatives and legislation aimed at regaining the public’s trust in vaccinations. More extensive studies are needed, especially in light of the prevalent misinformation about vaccines and the need to study actual vaccination uptake results in addition to intentions.

## Data Availability

Data are available upon reasonable request. Data are not publicly available due to the sensitive and personal nature of data and the collected information. Data may be available on request to authors, with restrictions following ethical approval. Please contact the corresponding author.
