# Supplementary material for: Human-centred design bolsters vaccine confidence in the Philippines: results of a randomised controlled trial
Source: BMJ Glob Health. 2023 Oct 21;8(10):e012613. doi: 10.1136/bmjgh-2023-012613 (PMC10603469; doi:10.1136/bmjgh-2023-012613)
Supplement: Supplementary data [file bmjgh-2023-012613supp001.pdf]

Project SALUBONG
Research Institute for Tropical Medicine, Philippines & University of Heidelberg, Germany

Person ID: |__|-|__|-|__||__||__|
Mun Brgy Participant No.

Project SALUBONG: Building Vaccine Confidence via Empathy and Narrative in the Philippines

SURVEY QUESTIONNAIRE FOR PARENTS or CARETAKERS OF CHILDREN UNDER-5 (PLEASE USE BLOCK LETTERS)

INSTRUCTION I: Read each question to the respondent. Put a cross (X) mark on the box corresponding to the answer given by the respondent under the "Options" column. If the response given is not in the given options, check "Others" and specify by writing the answer on the space provided.			
**Question No.**	**Questions**	**Options**	
**A. SURVEY IDENTIFICATION:** To be filled by the researcher.			
A1	Municipality Name		Code: |__|
A2	Barangay Name		Code: |__|
A3	Participant Number	|__||__||__|	
A4	Name of Interviewer		
A5	Date of Interview	|__||__|/|__||__|/ |__||__||__||__| (mm/dd/yyyy)	
**B. DEMOGRAPHIC INFORMATION OF THE RESPONDENT AND HOUSEHOLD CHARACTERISTICS**			
B1	First Name		
B2	Last Name		
B3	Sex	☐ 1 – Male ☐ 2 - Female	
B4	Birthday	|__||__|/|__||__|/ |__||__||__||__| (mm/dd/yyyy)	
B5	Respondent's Occupation	☐ 1- None	
		☐ 2- Self-employed	
		☐ 3- Housewife	
		☐ 4- Manual Laborer	
		☐ 5- Business	
		☐ 6- Farmer	
		☐ 7- Fisherman	
		☐ 8- Professional	
		☐ 99- Others, specify:	
B6	Respondent's highest educational attainment	☐ 1- Never attended school	
		☐ 2- Elementary	
		☐ 3- High school undergraduate	
		☐ 4- High school graduate	
		☐ 5- Vocational education	

Reñosa MDC, *et al. BMJ Glob Health* 2023; 8:e012613. doi: 10.1136/bmjgh-2023-012613

		☐ 6- College undergraduate
		☐ 7- College graduate
		☐ 8- Graduate studies
B7	How many children, including newborns, do you have or are currently taking care of in this family? |____|	

**Children**			**Is the child vaccinated according to the official vaccination schedule?** *(confirm with the vaccination card at the end of the interview)*	
**No.**	**First Name**	**Age of the child** (in years. If under 1-year-old, state age in months)	***Yes***	***No**, some of the vaccine have not been administered on schedule*
1				
2				
3				
4				
5				
6				
7				
8				

B8	Who is the primary decision-maker for health care concerns in the family?	☐ 1- Mother<br>☐ 2- Father<br>☐ 3- Grandmother<br>☐ 4- Other sibling<br>☐ 5- Other, (specify):<br>☐ 77- Prefer not to answer (skip to B9)<br>☐ 88- Don’t know (skip to B9)
B8.1	What is the age of the primary decision-maker in terms of health care? (in years)	|___| |___| years
B8.2	What is the highest educational attainment of the primary decision-maker in terms of health care?	☐ 1- Never attended school<br>☐ 2- Elementary<br>☐ 3- High school undergraduate<br>☐ 4- High school graduate<br>☐ 5- Vocational education<br>☐ 6- College undergraduate<br>☐ 7- College graduate

Project SALUBONG
Research Institute for Tropical Medicine, Philippines & University of Heidelberg, Germany

Person ID: |__|-|__|-|__||__||__|
Mun Brgy Participant No.

		☐ 8- Graduate studies
B9	Is your family part of the 4P's ("Pantawid Pamilyang Pilipino Program") of the government?	☐ 0 – No
		☐ 1 – Yes
		☐ 88 - Don't know
**C. SOURCES OF VACCINE-RELATED INFORMATION AND TRUST IN THESE SOURCES**		
C1	Have you received any information about vaccination?	☐ 0- No *(proceed to C2)*
		☐ 1- Yes *(proceed to C1.1)*
		☐ 88 - Don't know *(proceed to C2)*
C1.1	From whom, did you receive the information?	☐ 1- Friends and family members
		☐ 2- Barangay Health workers
		☐ 3- Midwife
		☐ 4- Nurse
		☐ 5- Doctor
		☐ 6- Newspapers
		☐ 7- Television
		☐ 8- Social Media (Facebook, Twitter, YouTube)
		☐ 99 – others, specify:
		☐ 88 - Don't know
C2	If you will receive information on vaccination, who is the source of information that you will trust the most?	☐ 1- Friends and family members
		☐ 2- Barangay Health workers
		☐ 3- Midwife
		☐ 4- Nurse
		☐ 5- Doctor
		☐ 6- Newspapers
		☐ 7- Television
		☐ 8- Social Media (Facebook, Twitter, YouTube)
		☐ 99 – others, specify: ____________________
		☐ 88 - Don't know
C3	If you will receive information on vaccination, who is the source of information that you will **NOT** trust the most?	☐ 1- Friends and family members
		☐ 2- Barangay Health workers
		☐ 3- Midwife
		☐ 4- Nurse
		☐ 5- Doctor
		☐ 6- Newspapers
		☐ 7- Television
		☐ 8- Social Media (Facebook, Twitter, YouTube)
		☐ 99 – others, specify: ____________________
		☐ 88 - Don't know
**D. PARENTS ATTITUDES ABOUT CHILDHOOD VACCINATION (PACV-15) (Adapted from Opel, et al. 2011)**		
D1		☐ 0 – No (proceed to D2)
		☐ 1 – Yes (proceed to D1.1)

Project SALUBONG
Research Institute for Tropical Medicine, Philippines & University of Heidelberg, Germany

Person ID: |__|-|__|-|__||__||__|
Mun Brgy Participant No.

	Have you ever **delayed** having your child get a shot (vaccine) for reasons other than illness of allergy?	
		☐ 88 - Don't know
D1.1	What was the reason/s for the delay? (multiple answers are allowed)	☐ 1 – Difficult of access to immunization center (proceed to D2)
		☐ 2 – No money for transportation (proceed to D2)
		☐ 3 – No one left at home to look after my other children (proceed to D2)
		☐ 4 – Vaccine is not safe (proceed to D2)
		☐ 5 – Vaccine is not effective (proceed to D2)
		☐ 6 – It was not my decision/ I wanted to go for vaccination but someone else disagreed (proceed to D1.2)
		☐ 99 – Others, specify: ________________________
D1.2	Who makes the decision about **NOT** getting vaccination?	☐ 1 – Husband
		☐ 2 – Mother/Wife
		☐ 3 – Other family members, specify: ___________
		☐ 77 – Don't want to answer
D2	Have you ever **decided NOT** to have your child get a shot (vaccine) for reasons other than illness or allergy?	☐ 0 – No (proceed to D3)
		☐ 1 – Yes (proceed to D2.1)
		☐ 88 - Don't know
D2.1	What was the reason/s you decided **NOT** to have your child be vaccinated? (multiple answers are allowed)	☐ 1 – Difficult of access to immunization center
		☐ 2 – No money for transportation
		☐ 3 – No one left at home to look after my other children
		☐ 4 – Vaccine is not safe
		☐ 5 – Vaccine is not effective
		☐ 6 – It was not my decision/ I wanted to go for vaccination but someone else disagreed
		☐ 99 – Others, specify: ________________________
D3	How sure are you that following the recommended shot (vaccine) schedule is a good idea for your child?<br><br>*Note: The respondent will be asked to choose from a 0-5 range category scale, with 0 as 'not all sure' to 10 as 'completely sure'.*	←——→<br>0 1 2 3 4 5<br>(not at all sure) (completely sure)
D4	Children get more shots (vaccination) than are good for them.	☐ 1- Strongly disagree
		☐ 2- Disagree
		☐ 3- Neither agree nor disagree
		☐ 4- Agree
		☐ 5- Strongly agree
D5	I believe that many of the illnesses that vaccinations prevent are severe.	☐ 1- Strongly disagree
		☐ 2- Disagree

Project SALUBONG
Research Institute for Tropical Medicine, Philippines & University of Heidelberg, Germany

Person ID: |__|-|__|-|__||__||__|
Mun Brgy Participant No.

		☐ 3- Neither agree nor disagree
		☐ 4- Agree
		☐ 5- Strongly agree
D6	It is better for my child to develop immunity by getting sick than to get a shot (vaccination).	☐ 1- Strongly disagree
		☐ 2- Disagree
		☐ 3- Neither agree nor disagree
		☐ 4- Agree
		☐ 5- Strongly agree
D7	It is better for my child to get fewer vaccines at the same time.	☐ 1- Strongly disagree
		☐ 2- Disagree
		☐ 3- Neither agree nor disagree
		☐ 4- Agree
		☐ 5- Strongly agree
D8	How concerned are you that your child might have serious side effect from a shot (vaccination)?	☐ 1- Very concerned
		☐ 2- Somewhat concerned
		☐ 3- Not sure
		☐ 4- Not too concerned
		☐ 5- Not concerned at all
D9	How concerned are you that any one of the childhood shots (vaccines) might not be safe?	☐ 1- Very concerned
		☐ 2- Somewhat concerned
		☐ 3- Not sure
		☐ 4- Not too concerned
		☐ 5- Not concerned at all
D10	How concerned are you that a vaccine might not be successful in preventing childhood diseases?	☐ 1- Very concerned
		☐ 2- Somewhat concerned
		☐ 3- Not sure
		☐ 4- Not too concerned
		☐ 5- Not concerned at all
D11	Overall, how hesitant about childhood shots (vaccination) would you consider yourself be?	☐ 1- Very hesitant
		☐ 2- Somewhat hesitant
		☐ 3- Not sure
		☐ 4- Not too hesitant
		☐ 5- Not hesitant at all
D12	I trust the information I receive about shots (vaccines) from health care worker	☐ 1- Strongly disagree
		☐ 2- Disagree
		☐ 3- Neither agree nor disagree
		☐ 4- Agree
		☐ 5- Strongly agree
D13	I am able to openly discuss my concerns about shots (vaccines) with my child's health care worker?	☐ 1- Strongly disagree
		☐ 2- Disagree
		☐ 3- Neither agree nor disagree
		☐ 4- Agree

Reñosa MDC, *et al. BMJ Glob Health* 2023; 8:e012613. doi: 10.1136/bmjgh-2023-012613

Project SALUBONG
Research Institute for Tropical Medicine, Philippines & University of Heidelberg, Germany

Person ID: |__|-|__|-|__||__||__|
Mun Brgy Participant No.

		☐ 5- Strongly agree
D14	All things considered, how much do you trust your child/s doctor?<br><br>*Note: The respondent will be asked to choose from a 0-5 range category scale, with 0 as 'not all sure' to 10 as 'completely sure'.*	← → 0 1 2 3 4 5<br>(not at all sure) (completely sure)
**E. FUTURE INTENT TO VACCINATE**		
E1	If I had another infant today, I would want him/her to get **all** the recommended vaccines.	☐ 0 – No<br>☐ 1 – Yes<br>☐ 88 - Don't know
E2	If I had another infant today, I will have him/her receive **only selected** pediatric vaccines.	☐ 0 – No (proceed to F1)<br>☐ 1 – Yes (proceed to E2.1)<br>☐ 88 - Don't know (proceed to F1)
E2.1	What vaccines you will not want to be administered?<br><br>Probe: If the mother cannot remember, interviewers will state the names of the childhood vaccines and let the mothers choose which they do not want to be administered.	Specify: __________
**F. PERCEPTION TO THE EDUCATIONAL MATERIALS**		
F1	I feel that the information I receive captures my concerns.	☐ 0 – No<br>☐ 1 – Yes<br>☐ 88 - Don't know
F2	What information did you receive?	☐ 1- vaccines are safe<br>☐ 2- vaccines are free<br>☐ 3- vaccines are effective<br>☐ 77 – cannot recall<br>☐ 99 – others, specify:
F3	In general sense, I feel that the people in the health care system respect my situation.	☐ 1- Strongly disagree<br>☐ 2- Disagree<br>☐ 3- Neither agree nor disagree<br>☐ 4- Agree<br>☐ 5- Strongly agree
F4		☐ 0 – No

Reñosa MDC, *et al. BMJ Glob Health* 2023; 8:e012613. doi: 10.1136/bmjgh-2023-012613

	I feel that I am warmly welcomed by health care workers in the health facilities.	☐ 1 – Yes<br>☐ 88 - Don't know
F5	I feel that agreeing to vaccines is a way to show my love for my children.	☐ 0 – No<br>☐ 1 – Yes<br>☐ 88 - Don't know
F6	I feel that agreeing to vaccines is a way to contribute in achieving healthy communities.	☐ 0 – No<br>☐ 1 – Yes<br>☐ 88 - Don't know
F7	I feel good when I think about vaccines.	☐ 0 – No<br>☐ 1 – Yes<br>☐ 88 - Don't know

POST-INTERVENTION SURVEY QUESTIONS

A. PARENTS ATTITUDES ABOUT CHILDHOOD VACCINATION (PACV-15) (Adapted from Opel, et al. 2011)		
A1	Have you ever **delayed** having your child get a shot (vaccine) for reasons other than illness of allergy?	☐ 0 – No<br>☐ 1 – Yes (proceed to A1.1)<br>☐ 88 - Don't know
A1.1	What was the reason/s for the delay?<br>(multiple answers are allowed)	☐ 1 – Difficult of access to immunization center (proceed to A2)<br>☐ 2 – No money for transportation (proceed to A2)<br>☐ 3 – No one left at home to look after my other children (proceed to A2)<br>☐ 4 – Vaccine is not safe (proceed to A2)<br>☐ 5 – Vaccine is not effective (proceed to A2)<br>☐ 6 – It was not my decision/ I wanted to go for vaccination but someone else disagreed (proceed to A1.2)<br>☐ 99 – Others, specify: _______________________
A1.2	Who makes the decision about **NOT** getting vaccination?	☐ 1 – Husband<br>☐ 2 – Other family members, specify: ____________<br>☐ 3 – Don't want to answer
A2	Have you ever **decided NOT** to have your child get a shot (vaccine) for reasons other than illness or allergy?	☐ 0 – No<br>☐ 1 – Yes (proceed to A2.1)<br>☐ 88 - Don't know
A2.1	What was the reason/s you decided **NOT** to have your child be vaccinated?<br>(multiple answers are allowed)	☐ 1 – Difficult of access to immunization center<br>☐ 2 – No money for transportation<br>☐ 3 – No one left at home to look after my other children<br>☐ 4 – Vaccine is not safe

Project SALUBONG
Research Institute for Tropical Medicine, Philippines & University of Heidelberg, Germany

Person ID: |__|-|__|-|__||__||__|
Mun Brgy Participant No.

		☐ 5 – Vaccine is not effective
		☐ 6 – It was not my decision/ I wanted to go for vaccination but someone else disagreed
		☐ 99 – Others, specify: ________________________
A3	How sure are you that following the recommended shot (vaccine) schedule is a good idea for your child?<br><br>*Note: The respondent will be asked to choose from a 0-5 range category scale, with 0 as 'not all sure' to 10 as 'completely sure'.*	←————————————→<br>0 1 2 3 4 5<br>(not at all sure) (completely sure)
A4	Children get more shots (vaccination) than are good for them.	☐ 1- Strongly disagree
		☐ 2- Disagree
		☐ 3- Neither agree nor disagree
		☐ 4- Agree
		☐ 5- Strongly agree
A5	I believe that many of the illnesses shots (vaccination) prevent are severe.	☐ 1- Strongly disagree
		☐ 2- Disagree
		☐ 3- Neither agree nor disagree
		☐ 4- Agree
		☐ 5- Strongly agree
A6	It is better for my child to develop immunity by getting sick than to get a shot (vaccination).	☐ 1- Strongly disagree
		☐ 2- Disagree
		☐ 3- Neither agree nor disagree
		☐ 4- Agree
		☐ 5- Strongly agree
A7	It is better for my child to get fewer vaccines at the same time.	☐ 1- Strongly disagree
		☐ 2- Disagree
		☐ 3- Neither agree nor disagree
		☐ 4- Agree
		☐ 5- Strongly agree
A8	How concerned are you that your child might have serious side effect from a shot (vaccination)?	☐ 1- Very concerned
		☐ 2- Somewhat concerned
		☐ 3- Not sure
		☐ 4- Not too concerned
		☐ 5- Not concerned at all
A9	How concerned are you that any one of the childhood shots (vaccines) might not be safe?	☐ 1- Very concerned
		☐ 2- Somewhat concerned
		☐ 3- Not sure
		☐ 4- Not too concerned

Reñosa MDC, *et al. BMJ Glob Health* 2023; 8:e012613. doi: 10.1136/bmjgh-2023-012613

Project SALUBONG
Research Institute for Tropical Medicine, Philippines & University of Heidelberg, Germany

Person ID: |__|-|__|-|__||__||__|
Mun Brgy Participant No.

		☐ 5- Not concerned at all
A10	How concerned are you that a shot (vaccine) might not prevent childhood diseases?	☐ 1- Very concerned<br>☐ 2- Somewhat concerned<br>☐ 3- Not sure<br>☐ 4- Not too concerned<br>☐ 5- Not concerned at all
A11	Overall, how hesitant about childhood shots (vaccination) would you consider yourself be?	☐ 1- Very hesitant<br>☐ 2- Somewhat hesitant<br>☐ 3- Not sure<br>☐ 4- Not too hesitant<br>☐ 5- Not hesitant at all
A12	I trust the information I receive about shots (vaccines) from health care workers	☐ 1- Strongly disagree<br>☐ 2- Disagree<br>☐ 3- Neither agree nor disagree<br>☐ 4- Agree<br>☐ 5- Strongly agree
A13	I am able to openly discuss my concerns about shots (vaccines) with my child's health care worker?	☐ 1- Strongly disagree<br>☐ 2- Disagree<br>☐ 3- Neither agree nor disagree<br>☐ 4- Agree<br>☐ 5- Strongly agree
A14	All things considered, how much do you trust your child/s doctor?<br><br>*Note: The respondent will be asked to choose from a 0-5 range category scale, with 0 as 'not all sure' to 10 as 'completely sure'.*	⟵⟶<br>0 1 2 3 4 5<br>(not at all sure) (completely sure)
**B. FUTURE INTENT TO VACCINATE**		
B1	If I had another infant today, I would want him/her to get **all** the recommended vaccines.	☐ 0 – No<br>☐ 1 – Yes<br>☐ 88 - Don't know
B2	If I had another infant today, I will have him/her receive **only selected** pediatric vaccines.	☐ 0 – No (proceed to C1)<br>☐ 1 – Yes (proceed to B2.1)<br>☐ 88 - Don't know (proceed to C1)
B2.1	What vaccines you will not want to be administered?<br><br>Probe: If the mother cannot remember, interviewers will state the names of the childhood vaccines and let the mothers choose which they do not want to be administered.	Specify: __________
**C. PERCEPTION TO THE EDUCATIONAL VIDEO**		

Survey Tool (English) version 6.0 15 July 2021 9

Project SALUBONG
Research Institute for Tropical Medicine, Philippines & University of Heidelberg, Germany

Person ID: |__|-|__|-|__||__||__|
Mun Brgy Participant No.

C1	I feel that the information I receive captures my concerns.	☐ 0 – No<br>☐ 1 – Yes<br>☐ 88 - Don't know
C2	What information did you receive?	☐ 1- vaccines are safe<br>☐ 2- vaccines are free<br>☐ 3- vaccines are effective<br>☐ 77 – cannot recall<br>☐ 99 – others, specify:
C3	In general sense, I feel that the people in the health care system respect my situation.	☐ 1- Strongly disagree<br>☐ 2- Disagree<br>☐ 3- Neither agree nor disagree<br>☐ 4- Agree<br>☐ 5- Strongly agree
C4	I feel that I am warmly welcomed by health care workers in the health facilities.	☐ 0 – No<br>☐ 1 – Yes<br>☐ 88 - Don't know
C5	I feel that agreeing to vaccines is a way to show my love for my children.	☐ 0 – No<br>☐ 1 – Yes<br>☐ 88 - Don't know
C6	I feel that agreeing to vaccines is a way to contribute in achieving healthy communities.	☐ 0 – No<br>☐ 1 – Yes<br>☐ 88 - Don't know
C7	I feel good when I think about vaccines.	☐ 0 – No<br>☐ 1 – Yes<br>☐ 88 - Don't know
**D. PERCEPTION TO THE INTERVENTION (Specific for the Intervention Group)**		
D1	I feel that the family concerns in the video relates my own concerns regarding childhood vaccines	☐ 0 – No (proceed to D1.2)<br>☐ 1 – Yes (proceed to D1.1)<br>☐ 88 - Don't know (proceed to D2)
D1.1	In the video you just watched, who do you much relate to?<br><br>*(multiple answers applicable)*	☐ 0 – None<br>☐ 1 – Pat (who agrees with the decision of household head)<br>☐ 2 – Nanang (who believes in herbal remedies)<br>☐ 3 – Nena (who believes with rumors in social media)<br>☐ 4 – Husband of Nena (who believes that vaccines has side effects and caused more harm to children)<br>☐ 5 – Sally (who expressed competing time demands)<br>☐ 6 – Doctor Beth
D1.2	In the video you just watched, who do you much **NOT** relate to?<br><br>*(multiple answers applicable)*	☐ 0 – None<br>☐ 1 – Pat (who agrees with the decision of household head)<br>☐ 2 – Nanang (who believes in herbal remedies)<br>☐ 3 – Nena (who belieSurve verThe ves with rumors in social media)

		☐ 4 – Husband of Nena (who believes that vaccines has side effects and caused more harm to children)
		☐ 5 – Sally (who expressed competing time demands)
		☐ 6 – Doctor Beth
D2	The video I just watched gives a clear message about the importance of childhood vaccination.	☐ 0 – No
		☐ 1 – Yes
		☐ 88 - Don't know
D3	Overall, the video I just watched was appealing and engaging.	☐ 0 – No
		☐ 1 – Yes
		☐ 88 - Don't know

Thank you for participating!
